# A response prediction model for taxane, cisplatin, and 5-fluorouracil chemotherapy in hypopharyngeal carcinoma

**DOI:** 10.1038/s41598-018-31027-y

**Published:** 2018-08-23

**Authors:** Qi Zhong, Jugao Fang, Zhigang Huang, Yifan Yang, Meng Lian, Honggang Liu, Yixiang Zhang, Junhui Ye, Xinjie Hui, Yejun Wang, Ying Ying, Qing Zhang, Yingduan Cheng

**Affiliations:** 1 Department of Otolaryngology Head and Neck Surgery, Beijing Tongren Hospital, Capital Medical University, Beijing, China; 2 Beijing Institute of Otolaryngology, Beijing, China; 3 Key Laboratory of Otolaryngology Head and Neck Surgery, Capital Medical University, Ministry of Education, Beijing, China; 4 Beijing Key Laboratory of Head and Neck Molecular Diagnostic Pathology, Beijing, 100730 China; 5 Department of Urology, The Second Affiliated Hospital of Jinan University, Shenzhen People’s Hospital, Shenzhen, Guangdong, China; 6 Neurontechnology, Shenzhen, Guangdong, China; 7 Department of Cell Biology and Medical Genetics, Shenzhen University Medical School, Shenzhen, 518060 China; 8 Department of Physiology, School of Basic Medical Sciences, Shenzhen University Health Sciences Center, Shenzhen, 518060 China

## Abstract

Head and neck squamous cell carcinoma (HNSCC) is the sixth most common cancer worldwide. The five-year survival rate of HNSCC has not improved even with major technological advancements in surgery and chemotherapy. Currently, docetaxel, cisplatin, and 5-fluoruracil (TPF) treatment has been the most popular chemotherapy method for HNSCC; but only a small percentage of HNSCC patients exhibit a good response to TPF treatment. Unfortunately, at present, no reasonably effective prediction model exists to assist clinicians with patient treatment. For this reason, patients have no other alternative but to risk neoadjuvant chemotherapy in order to determine their response to TPF. In this study, we analyzed the gene expression profile in TPF-sensitive and non-sensitive patient samples. We identified a gene expression signature between these two groups. We further chose 10 genes and trained a support vector machine (SVM) model. This model has 88.3% sensitivity and 88.9% specificity to predict the response to TPF treatment in our patients. In addition, four more TPF responsive and four more TPF non-sensitive patient samples were used for further validation. This SVM model has been proven to achieve approximately 75.0% sensitivity and 100% specificity to predict TPF response in new patients. This suggests that our 10-genes SVM prediction model has the potential to assist clinicians to personalize treatment for HNSCC patients.

## Introduction

Head and neck squamous cell carcinoma (HNSCC) is the sixth most common cancer worldwide^1^. Risk factors for HNSCC include tobacco and alcohol consumption, as well as human papilloma virus (HPV) infection^2^. In the United States (U.S.) alone, there are approximately 40,000 new cases diagnosed annually and approximately 7,890 of those cases led to death^3^. Despite major advancements in surgery and chemotherapy, the five- year survival rate of HNSCC has not improved^4^. In the past, surgeons have sometimes had to remove the functional organ for better prognosis. Currently, in order to improve the quality of life of HNSCC patients, especially for laryngeal, oro- and hypopharyngeal cancer, preserving a functional organ constitutes a focus for surgeons and oncologists^5^. With this aim, efficient chemotherapy treatment has become even more critical.

Based on clinical studies, results suggest that chemotherapy is the most beneficial treatment for HNSCC^6,7^. The prediction of patients’ response from induction chemotherapy, based on biomarkers, avoids the toxic effect of ineffective chemotherapy, as well as future delays of other therapeutic options. In addition, the development of personalized medicine strategies benefits both patients and countries. Several biomarkers possess potential clinical significance to predict therapy response^8^. For example, low expression of the epidermal growth factor receptor (EGFR) is a potential biomarker in predicting chemotherapy response in HNSCC^8–11^. However, lower expression of EGFR failed to correlate with better survival rates in response to combined cisplatin and EGF inhibitor cetuximab^12^. Although lower CCND1 was an independent predictor of chemotherapy response and survival in induction groups^8,13^ multivariate analysis indicated that CCND1 provides no prognostic or predictive benefit in oral or oropharyngeal SCC^14^.

Currently, a single predictor marker is not sufficiently effective to predict chemotherapy response. Moreover, combined therapy is more popular and effective than single drug treatments. For instance, the combination of cisplatin and 5-fluoruracil (PF) induction chemotherapy has proven to be a successful treatment for HNSCC. However, with the increase of relevant clinical studies, TPF treatment has been more widely utilized with patients, since TPF has better survival and organ preservation rates compared to PF therapy in locally advanced laryngeal, oro- and hypopharyngeal cancer^15,16^. Even though some biomarkers have been reported to predict the response to induction chemotherapy, microarray-based gene expression profiles for predicting TPF chemotherapy have not yet been well investigated. In addition, there is currently no prediction model for TPF treatment.

The prediction of chemotherapy response, with a set of genes, has been widely used in breast cancer. Twenty-one gene expression levels were utilized as a guideline for adjuvant chemotherapy in hormone-receptor–positive, HER2-negative, and axillary node–negative breast cancer^15–17^. This breast cancer model constitutes a good example of personalized medicine, and has also saved at least USD 300 million of unnecessary chemotherapy costs in the U.S. Here, we used microarray-based gene expression profiles to identify the gene signature that related to TPF response. We first identified a group of genes, which could predict chemotherapy response in HNSCC, in a SVM model. We further validated this gene signature with more patient samples. Our study provides a set of potential biomarkers to predict patient response to TPF treatment, as well as the possible benefits of: 1) avoiding toxic effects of ineffective chemotherapy; 2) avoiding delays for other therapeutic options; and 3) minimizing the cost of treatment^18^.

## Results

### Patients’ clinical characteristics

Here, a total of 29 patients were enrolled in this study. Among them, 16 patients were considered sensitive to TPF treatment, and 13 patients as non-sensitive to TPF treatment. We classified the sensitive and non-sensitive groups by patients’ response to chemotherapy. If tumor volume decreased approximately 70% after chemotherapy, we considered it as a chemotherapy-sensitive one. If tumor volume decreased less than approximately 25%, we considered it as a chemotherapy non-sensitive one. The decreased volume between 25–75% was excluded in our study. Then, we randomly chose the sensitive and non-sensitive samples for our study. The first 21 samples (twelve sensitive and nine non-sensitive) were used to set up the prediction model for TPF treatment (Table 1), and the second group (four sensitive and four non-sensitive) was used to validate our prediction model (Table 1). Here, we excluded some factors that have prediction potential for TPF treatment, i.e., gender, age, primary site, stage, differentiation degree, gastrointestinal reaction, myelosuppression, family history, smoking history, and alcohol intake (Table 2).

**Table 1:** Clinical characteristics of the first group of patients.

No.	Gender	Age	Primary site	TNM	Stage	Differentiation degree	Cycles of chemotherapy	Chemotherapy regimen	Efficacy
Drug-sensitive group									
1	M	69	left pyriform sinus	T4aN2M0	IVA	high	3	TPF	PR
2	M	69	retropharyngeal wall	T4aN2M0	IVA	high	2	TPF	PR
3	M	49	aryepiglottic fold	T3N2M0	IVA	moderate	2	TPF	CR
4	M	62	right pyriform sinus	T4aN2M0	IVA	poor	2	TPF	PR
5	M	60	retropharyngeal wall	T4bN2M0	IVA	moderate	2	TPF	PR
6	M	69	right pyriform sinus	T4aN0M0	IVA	moderate	2	TPF	PR
7	M	49	aryepiglottic fold	T4aN2M0	IVA	moderate	2	TPF	PR
8	M	44	left pyriform sinus	T4aN2M0	IVA	poor	2	TPF	PR
9	M	60	left pyriform sinus	T3N1M0	III	moderate	3	TPF	PR
10	M	48	left pyriform sinus	T4bN2cM0	IVB	high	2	TPF	PR
11	M	53	right pyriform sinus	T4aN0M0	IVA	high	2	TPF	PR
12	M	45	left pyriform sinus	T2N2M0	IVA	moderate or poor	2	TPF	PR
**13**	**F**	**48**	**left pyriform sinus**	**T4bN2M0**	**IVB**	**moderate**	**2**	**TPF**	**PR**
**14**	**M**	**59**	**retropharyngeal wall**	**T3N0M0**	**III**	**high**	**2**	**TPF**	**PR**
**15**	**M**	**57**	**right pyriform sinus involving oropharynx**	**T4bN0M0**	**IVB**	**moderate**	**2**	**TPF**	**PR**
**16**	**M**	**64**	**right pyriform sinus**	**T4aN2M0**	**IVA**	**poor**	**2**	**TPF**	**PR**
Drug non-sensitive group									
17	M	65	left pyriform sinus	T4aN2M0	IVA	high	2	TPF	SD
18	M	45	left pyriform sinus	T2N3M0	IVB	high	2	TPF	PD
19	M	69	left pyriform sinus	T3N2M0	IVA	moderate	2	TPF	SD
20	M	71	left pyriform sinus	T4aN2M0	IVA	poor	2	TPF	SD
21	M	69	right pyriform sinus	T2N1M0	III	high	2	TPF	SD
22	M	71	lateral pharyngeal wall	T4aN0M0	IVA	high	2	TPF	SD
23	M	43	postcricoid	T4aN2M0	IVA	moderate	2	TPF	SD
24	M	57	right pyriform sinus	T4bN3M1	IVC	high	2	TPF	SD
25	M	43	right pyriform sinus	T4bN2M1	IVC	poor	3	TPF	SD
**26**	**M**	**58**	**postcricoid**	**T4aN3M0**	**IVB**	**poor**	**2**	**TPF**	**SD**
**27**	**M**	**63**	**left pyriform sinus**	**T4aN1M0**	**IVA**	**moderate**	**3**	**TPF + Nimotuzumab**	**SD**
**28**	**M**	**73**	**right pyriform sinus**	**T3N0M0**	**III**	**moderate**	**2**	**TPF**	**SD**
**29**	**M**	**61**	**retropharyngeal wall**	**T4aN2M0**	**IVA**	**high**	**2**	**TPF**	**SD**

CR, complete response: Disappearance; confirmed at four weeks; PR, partial response: 50% decrease; confirmed at four weeks; SD, stable disease: Neither PR nor PD criteria met; PD, progressive disease: 25% increase; no CR, PR, or SD documented before increased disease. 13–16 are the second batch of the drug-sensitive group (bold). 26–29 are the second batch of the drug non-sensitive group (bold).

**Table 2:** Characteristics of patients.

Characteristics	Response to chemotherapy				P value
	Drug non-sensitive		Drug-sensitive		
	No. of patients (n = 13)	%	No. of patients (n = 16)	%	
**Gender**					1
male	13	100	15	93.8	
female	0	0	1	6.3	
**Age**					0.16
41–60	5	38.5	11	68.8	
61–80	8	61.5	5	31.2	
**Tumor location**					0.991
left pyriform sinus	5	38.5	6	37.5	
right pyriform sinus	4	30.8	5	31.3	
retropharyngeal wall	1	7.7	3	18.8	
lateral pharyngeal wall	1	7.7	0	0	
aryepiglottic	0		2	12.5	
postcricoid	2	15.4	0	0	
**Family history**	1	7.7	1	6.3	0.879
**Stage**					0.983
III	2	15.4	2	12.5	
IVA	7	53.8	11	68.8	
IVB	2	15.4	3	18.8	
IVC	2	15.4	0		
**Differentiation degree**					0.690
poor	3	23.1	4	25.0	
moderate	4	30.8	7	43.8	
high	6	46.2	5	31.3	
**Gastrointestinal reaction**					0.314
0	4	30.8	9	56.3	
I	9	69.2	6	37.5	
III	0		1	6.3	
**Myelosuppression**					0.789
0	9	69.2	10	62.5	
I	3	23.1	2	12.5	
II	1	7.7	3	18.8	
III	0	0	1	6.3	
**Alcohol**					0.704
no	2	15.4	2	12.5	
occasional	1	7.7	3	18.8	
yes	10	76.9	11	68.8	
**Smoking**					0.405
smoking	10	76.9	13	81.3	
no smoking	3	23.1	3	18.8	

### Gene expression profiles

Firstly, a microarray was performed for the first group patients’ samples. The data were analyzed by a standard approach^19^. The second group was used as a validation for our previous experiment, and was analyzed in the same way.

### A prediction model for TPF treatment in HNSCC

**Figure 1:**
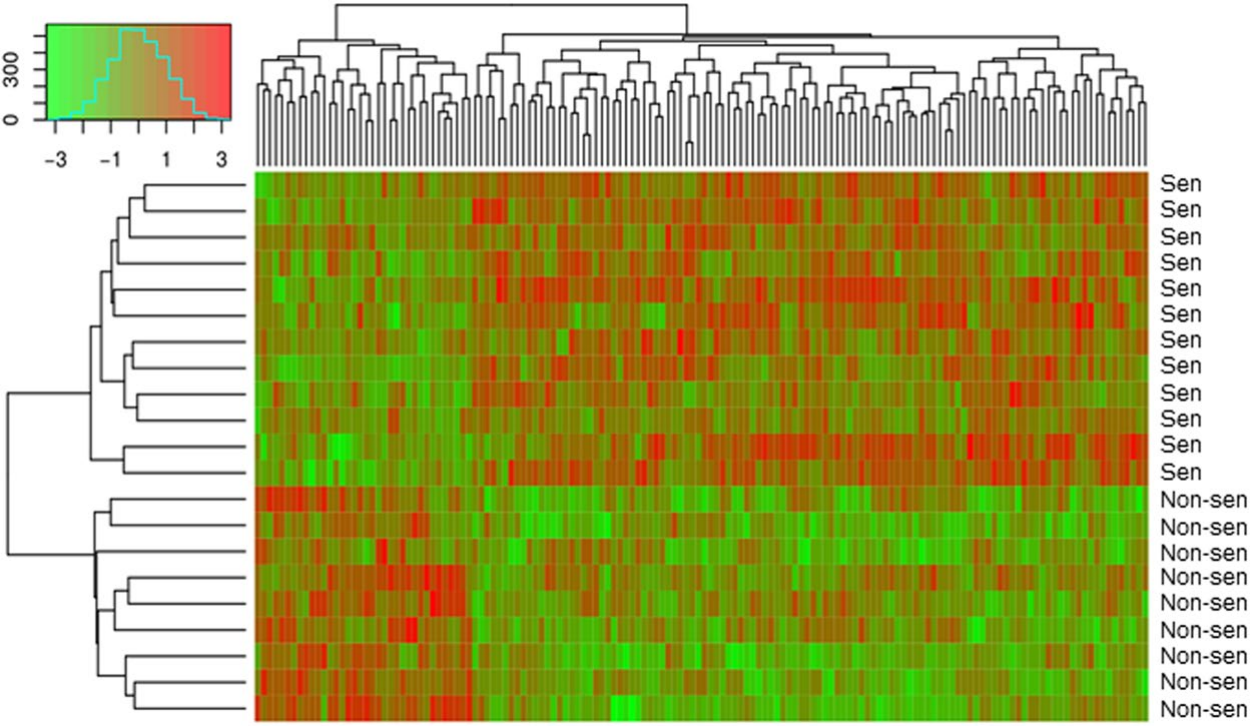
Heatmap of the first group of samples. Twenty-one samples were included in the first study. The rows represent the samples; the text at the right of each row describes the TPF sensitivity (Sen) or non-sensitivity (Non-sen) sample. The columns represent different expressed genes (DEGs). The color shows the expression levels of DEGs in the samples (z-score normalized by columns).

Our hypothesis is that the mRNA expression profiles of the treatment-sensitive patients are distinct from those of the treatment non-sensitive patients. To prove this, we firstly used the limma package to select a set of differential expression genes (p value ≤ 0.01); and then generated the heatmap figure based on these genes (Fig. 1). All of the samples were clustered into two groups, i.e., the treatment-sensitive and treatment non-sensitive group. Based on the expression levels, the genes were also clustered into two groups. A set of genes was highly expressed in the treatment-sensitive samples, and another set of genes was highly expressed in the treatment non-sensitive samples. These results motivated us to build a model that can predict the response of the patient to the treatment based on his or her mRNA expression levels.

**Figure 2:**
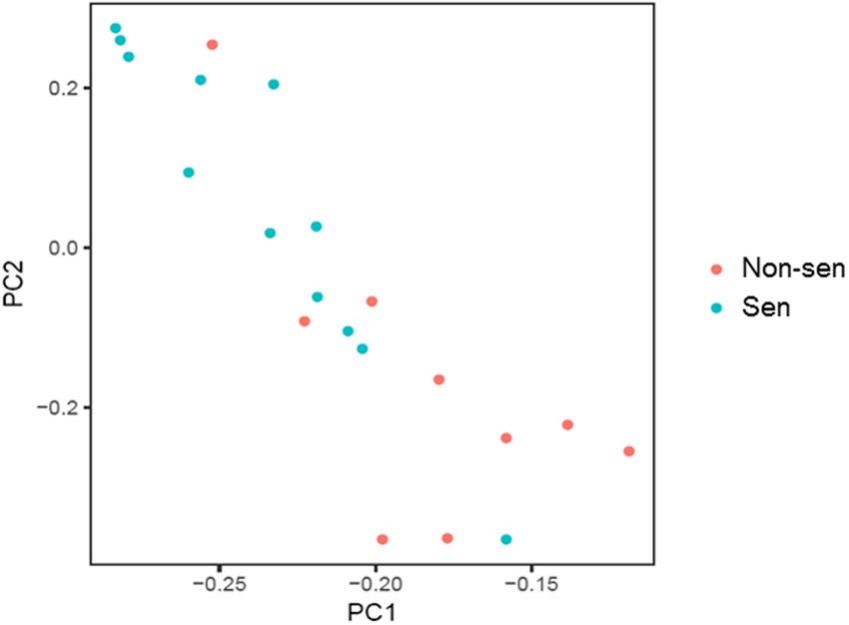
Principal components analysis. X-axis: the first principal component; y-axis: the second principal component. The scores of the first (PC1) and second (PC2) principle components were plotted. The color shows the category (Sen or Non-sen) of the sample.

As described in the Methods section, we trained a SVM model and obtained 722 genes. To avoid the false-positive genes, based on our clinical knowledge, we selected 10 genes that are known to relate to TPF or be potentially involved in the treatment pathway. These genes are GATS, PRIC285, ARID3B, ASNS, CXCR1, FBN2, INMT, MYOM3, SLC27A5, and STC2. The gene list is found in Table 3. We estimated the performance of the model using the one-leave-out method based on the old data. The sensitivity and specificity of this model are 88.3% and 88.9%, respectively (Fig. 2).

**Table 3:** A list of genes that could predict the response in our SVM model.

No.	Gene title	Gene symbol	Gene function	Gene fold change log2 (sensitive/non-sensitive)
1	GATS	GATS	stromal antigen 3 opposite strand	0.874254
2	helicase with zinc finger 2	PRIC285	nuclear transcriptional co-activator for peroxisome proliferator activated receptor alpha	−0.72736
3	AT-rich interaction domain 3B	ARID3B	embryonic patterning, cell lineage gene regulation, cell cycle control, transcriptional regulation and possibly in chromatin structure modification	0.457583
4	asparagine synthetase (glutamine-hydrolyzing)	ASNS	synthesis of asparagine	0.554069
5	C-X-C motif chemokine receptor 1	CXCR1	transduces the signal through a G-protein activated second messenger system	−0.62679
6	fibrillin 2	FBN2	a component of connective tissue microfibrils, and may be involved in elastic fiber assembly	1.747669
7	indolethylamine N-methyltransferase	INMT	detoxification of selenium compounds	−0.44659
8	myomesin 3	MYOM3	link the intermediate filament cytoskeleton to the M-disk of the myofibrils in striated muscle	−0.83787
9	solute carrier family 27 (fatty acid transporter), member 5	SLC27A5	capable of activating very long-chain fatty-acids containing 24- and 26-carbons	0.902843
10	stanniocalcin 2	STC2	regulation of renal and intestinal calcium, and phosphate transport, cell metabolism, or cellular calcium/phosphate homeostasis	1.031989

### Further validation with eight more patients’ samples

We trained a 10-genes prediction model with 88.3% sensitivity and 88.9% specificity. Then, we aimed to determine if this model could be validated by more samples. We further checked the gene expression with eight more patients’ samples by microarray. Among them, four samples were sensitive and four samples were non-sensitive samples. We used our model to test those samples and found that our model has good performance. For the new data, we determined the sensitivity and specificity of our model as 75.0% and 100%, respectively. This suggested that our model is sufficient to predict TPF treatment performance in HNSCC.

### Immunohistochemistry (IHC)

**Figure 3:**
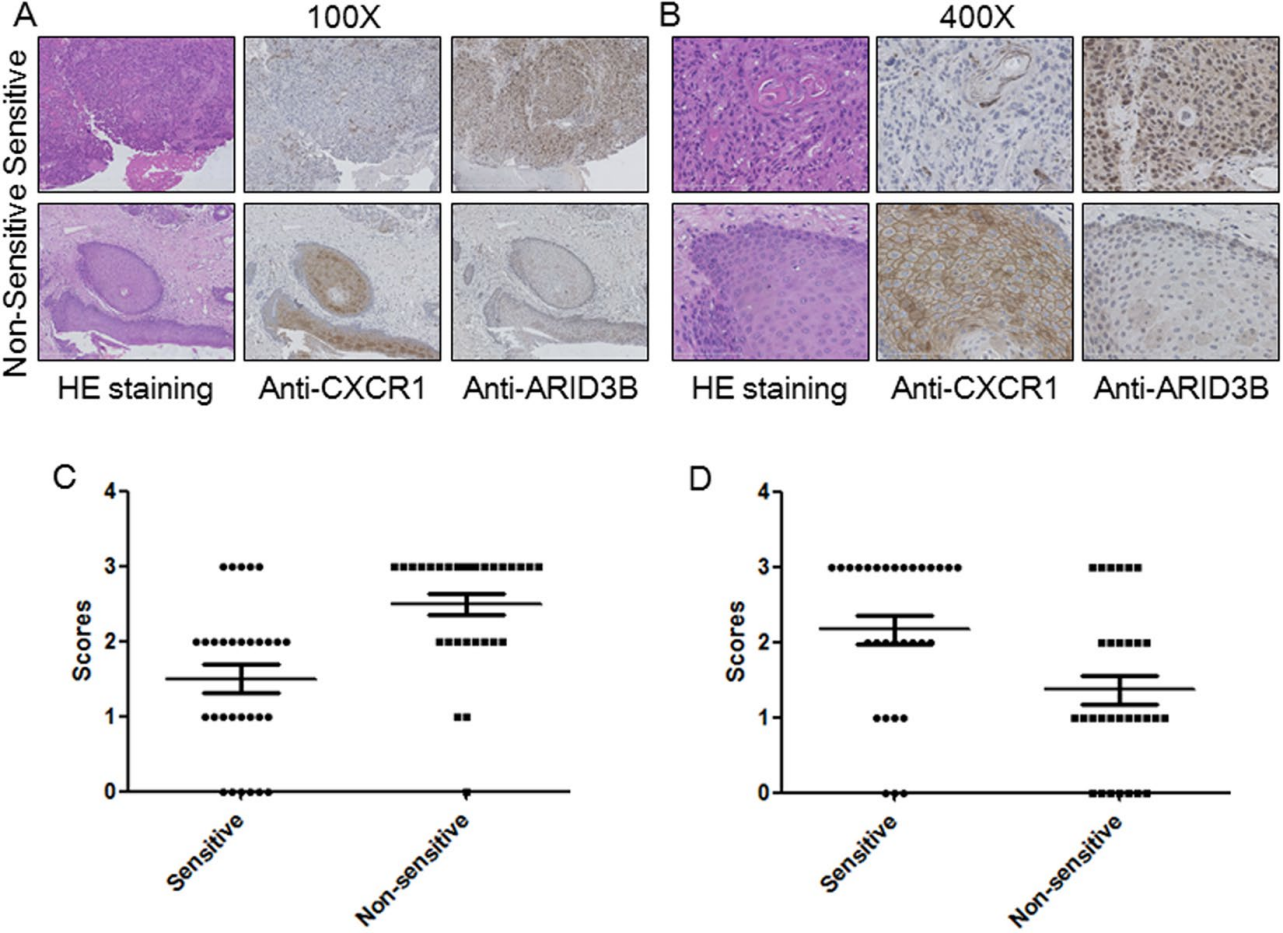
Immunostaining of candidate genes. (**A**,**B**) Presentative IHC images of CXCR1 and ARID3B with different (**A**) 100X; (**B**) 400X. (**C**) Statistical analysis of the immunohistochemistry results for CXCR1 and ARID3B. Student t-test, *p < 0.05, **p < 0.01.

In order to confirm the protein levels of the candidate genes in sensitive and non-sensitive patients, we performed IHC for CXCR1 and ARID3B. We found that CXCR1 was upregulated in non-sensitive patients’ samples and ARID3B was downregulated in non-sensitive patients’ samples (Fig. 3). These findings correlated well with our microarray results.

## Discussion

Although TPF treatment is currently the most popular therapy for HNSCC patients, no effective prediction model exists to avoid unnecessary treatment and aid in relieving patients’ pain. Here, we identified the gene signature in TPF-sensitive and non-sensitive patients. In order to predict TPF treatment response, we selected 10 genes that exhibit good correlation with treatment. Based on these 10 genes, a model was set up to predict TPF treatment in HNSCC patients. This model had 88.3% sensitivity and 88.9% specificity for prediction. We further validated this model with eight additional samples. Our model had 75.0% sensitivity and 100% specificity in those samples, which indicated that our prediction model is sufficient to predict the response to TPF treatment.

A major problem with the treatment options for HNSCC is that clinicians do not know the treatment response of each patient prior to chemotherapy, which will delay the best choice of treatment for patients. The purpose of personalized medicine is to separate patients into different groups. In this way, based on patients’ predicted response or disease risk, medical decisions, practices, interventions, and/or products are optimally tailored to the individual patient. Here, we developed a model to predict chemotherapy response based on a 10-genes model, which may be beneficially utilized for personalized medicine in HNSCC patients.

The 10-genes model is based on the gene expression of GATS, PRIC285, ARID3B, ASNS, CXCR1, FBN2, INMT, MYOM3, SLC27A5, and STC2. Among these 10 genes, some have been reported to be involved in cell cycle, apoptosis, and drug metabolism. For instance, ARID3B belongs to a subfamily of ARID (AT-rich interaction domain) transcription factors. It could increase tumor growth *in vivo*, inducing expression of genes associated with metastasis and cancer stem cells^20–22^. ASNS (asparagine synthetase, ASNS) gene encodes an enzyme that catalyzes glutamine- and ATP-dependent conversion of aspartic acid to asparagine, and its expression is associated with chemotherapy resistance and prognosis in several human cancers^23–25^. CXCR1 is a member of the G-protein-coupled receptor family and functions as a receptor for interleukin 8. CXCR1 promotes tumor growth, invasion, inflammation and metastasis, and the knockdown of CXCR1 will enhance the efficacy of chemotherapy^26–29^. FBN2 is a tumor suppressor in cancer and is frequently silenced by promoter methylation^30,31^. STC2 is an oncogenic gene that promotes cancer metastasis and epithelial-mesenchymal transition in cancers^32,33^, and the induction of oxaliplatin resistance in colorectal cancer^34^. These studies indicate that the genes in our prediction model possess known functions in cancer progression and chemotherapy.

Currently, our prediction model has approximately 75.0% sensitivity and 100% specificity for chemotherapy response prediction. Our future work will use more samples to continue validating our prediction model. Our work has the potential to assist clinicians to predict the treatment response of HNSCC patients prior to chemotherapy and enable clinicians to personalize medicine for each patient.

## Methods

### Patients’ samples

All patients’ samples were collected before chemotherapy in Beijing Tongren Hospital from patients who had given consent. The first batch was collected from June, 2013 to February, 2014. The second batch was collected from January, 2015 to June, 2015. All experimental protocols were approved by the Ethics Committee of Beijing Tongren Hospital, and all experiments were carried out in accordance with relevant guidelines and regulations of the same Ethics Committee. All patients were diagnosed with advanced hypopharyngeal carcinoma before surgery or treatment. Patient samples were collected by biopsy. Fresh samples were snap-frozen in liquid nitrogen and stored at −80 °C until RNA extraction.

### Patient treatment

Patients were treated with TPF chemotherapy. The treatment procedure was as described previously^35–37^. Briefly, we performed three 21-day cycles of induction chemotherapy with docetaxel (75 mg/m^2^ continuous i.v. infusion for more than 3 h on day 1), cisplatin (30 mg/m^2^), and 5-FU (500 mg/m^2^)^37^. We chose 29 samples for our studies, i.e., 16 patients that responded well to TPF chemotherapy and 13 patients that ranged from no response to poor response to TPF treatment. These samples were then divided into two groups based on the new Response Evaluation Criteria in Solid Tumors^38^. The first group had 12 sensitive patients and nine non-sensitive patients. The second group had four sensitive patients and four non-sensitive patients. All patients’ information is summarized in Table 1 and Supplemental Table 1. The patients’ gender, age, primary site, TNM, stage, cycle of chemotherapy, chemotherapy regimen, gastrointestinal reaction, myelosuppression efficacy, family history, precancerosis, gastroesophageal reflux, smoking history, and alcohol intake were included in our study.

### RNA extraction

In total, 16 TPF-sensitive patient samples and 13 TPF non-sensitive patient samples were chosen for microarray study. RNA was extracted from the samples by Qiagen RNeasy Mini Kit. Gene expression profiles were then analyzed by microarray (Chip type Human-HT-12-V4, illumina).

### Data analysis, gene selection, and model construction

The microarray data from the CEL files were normalized using RMA. We used the genes that were significantly differentially expressed (DEs) in the TPF-sensitive group or in the TPF non-sensitive group as features to construct the prediction model. Because our sample size was small, to robustly estimate our model and pick up the DEs as much as possible, we built an iterative one-leave-out process. We removed one patient from the first batch of data, and inferred the DEs based on the remaining data. The expression levels of these DEs were used to construct the prediction model (SVM). We then predicted the group of leave-out patients. We repeated the two steps until all 21 patients in the first batch had been removed once. So, we could obtain the average of the sensitivity and specificity based on the first batch of data alone. Here, we used the limma package to find the DEs and the required p-value < 0.01. Then, we collected all DEs from the 21 rounds, and totally, we obtained 722 genes. All genes were listed as Supplemental Table 2. To avoid false-positive genes, based on our clinical knowledge, we selected 10 genes that are known to relate to TPF or be potentially involved in the treatment pathway. The expression levels of the selected genes in the first batch of data were used to train the support vector machine model and predict the group of patients in the second batch of data independently.

### Estimation of predictive power for the new data

We used two values to estimate the performance of the predictive model based on the new data: Sensitivity: True Positive/(True Positive + False Negative); Specificity: True Negative/(True Negative + False Positive). Here, “positive” means that the individual patient is sensitive to the treatment; “negative” means that the individual patient is not sensitive to the treatment. With the first-round data, we applied the one-leave-out cross validation method to estimate the performance of the SVM model. For the second-round data, we trained SVM using the first-round data with all 21 samples, and calculated the sensitivity and specificity.

### Patient samples and IHC

Thirty TPF-sensitive patient samples and 30 TPF non-sensitive hypopharyngeal carcinoma patient samples were collected in Beijing Tongren Hospital with patients’ permission. IHC was performed on 4-µm sections of formalin-fixed, paraffin-embedded human hypopharyngeal carcinoma tissues. Sections were deparaffinized, rehydrated, and subjected to heat-induced antigen retrieval. After incubation with blocking solution, sections were incubated with anti-CXCR1 antibody (Abcam) or ARID3B antibody (Abcam) for 1 h, biotinylated secondary antibody for 30 min, and then streptavidin horseradish peroxidase for another 10 min. Sections were developed with 3,3′-diaminobenzidine chromogen and further stained with hematoxylin. An H-score was assigned to each tissue based on the product of staining intensity ((−), no staining; (+), weak; (++), moderate; and (+++), strong) and the percentage of stained cells (0–0%, 1–1% to 30%, 2–31% to 70%, and 3–71% to 100%).

### Statistical analysis

The data are presented as mean values ± standard deviation (SD) and statistically compared between groups using one-way analysis of variance followed by Student’s t-test. The significance of the variables was tested using a multivariate Cox’s regression model and a logistic regression model. A p-value of <0.05 was considered statistically significant.

## Electronic supplementary material

Supplementary table 1

Supplementary table 2
