# Supplementary material for: A response prediction model for taxane, cisplatin, and 5-fluorouracil chemotherapy in hypopharyngeal carcinoma
Source: Sci Rep. 2018 Aug 23;8:12675. doi: 10.1038/s41598-018-31027-y (PMC6107664; doi:10.1038/s41598-018-31027-y)
Supplement: Supplementary file 1 — Supplementary table 1 [file 41598_2018_31027_MOESM1_ESM.doc]

**A response prediction model for taxane, cisplatin and 5-fluorouracil chemotherapy in hypopharyngeal carcinoma**

Qi Zhong^1,2,3^, Jugao Fang^1,2,3*^, Zhigang Huang^1,2,3^, Yifan Yang^1,2,3^, Meng Lian^1,2,3^, Honggang Liu^4^, Yixiang Zhang^5^, Junhui Ye^6^, Xinjie Hui^7^, Yejun Wang^7^, Ying Ying^8^, Qing Zhang^6*^ & Yingduan Cheng^5*^

**Supplemental table 1**: Clinical characteristics of the patients. 1-12, first drug sensitive group; 13-16, second drug sensitive group; 17-25, first drug non-sensitive group; 26-29, second drug non-sensitive group.

|  | No | gastrointestinal reaction | myelosuppression | family history | precancerosis | gastroesophageal reflux | smoking | alcohol intake |
| --- | --- | --- | --- | --- | --- | --- | --- | --- |
| Drug sensitive group | 1 | I | 0 | no | no | no | 40/d*50y | 50g/d*50y |
|  | 2 | 0 | II | no | no | no | 20/d*45 | 250g/d*45 |
|  | 3 | 0 | 0 | no | no | no | 20/d*30y | 150g/d*30y |
|  | 4 | III | 0 | no | no | no | 20/d*40y | 125g/d*40y |
|  | 5 | 0 | II | no | no | no | 40/d*20y | 75g/d*20y,quit for 12y |
|  | 6 | 0 | 0 | no | no | no | 20/d*50y | occasional |
|  | 7 | I | 0 | no | no | no | 20/d*20y | 150g/d*20y |
|  | 8 | I | 0 | father died of stomach cancer | no | no | 5-10/d*20y | 75g/d*20y |
|  | 9 | I | I | no | no | no | 15/d*40y | 125g/d*35y |
|  | 10 | 0 | 0 | no | no | no | 10/d*5y | 75g/d*25y |
|  | 11 | 0 | 0 | no | no | no | 20/d*30y | 250g/d*30y |
|  | 12 | I | III | no | no | no | no | occasional |
|  | 13 | 0 | 0 | no | no | no | no | no |
|  | 14 | 0 | 0 | no | Vocal cord carcinoma have laser treatment for five years | no | no | no |
|  | 15 | I | I | no | no | no | 20/d*30y | 200g/d*30y |
|  | 16 | 0 | II | no | no | no | 20/d*30y | occasional |
| Drug non-sensitive group | 17 | I | 0 | no | no | no | 20/d*40y | 75g/d*20y |
|  | 18 | 0 | 0 | no | no | no | 30/d*20y | 75g/d*20y |
|  | 19 | I | 0 | no | no | no | 20/d*50y | 100g/d*50y |
|  | 20 | I | I | no | no | no | 20/d*50y | 125g/d*50y |
|  | 21 | I | 0 | no | no | no | 20/d*20y, quit for 5 years | 125g/d*20y |
|  | 22 | I | 0 | no | no | no | no | no |
|  | 23 | 0 | 0 | no | no | no | 10/d*20y | 100g/d*20y |
|  | 24 | I | 0 | no | no | no | 10/d*40y | 60g/d*40y |
|  | 25 | I | I | no | no | no | 20/d*30y | 250g/d*20y |
|  | 26 | I | I | no | no | no | 20/d*20y, quit for 4 years | occasional |
|  | 27 | 0 | 0 | no | no | no | 20/d*40y | 75g/d*40y |
|  | 28 | I | II | mother- esophagus cancer and laryngeal cancer elder sister- esophagus cancer young sister- esophagus cancer | no | no | 10/d*50y | no |
|  | 29 | 0 | 0 | no | no | no | 30/d*40y | 175g/d*40y |
